# Listeriosis in a peri-urban area: Cultural and molecular characterization of *Listeria monocytogenes* isolated from encephalitic goats

**DOI:** 10.14202/vetworld.2020.1743-1749

**Published:** 2020-09-02

**Authors:** Nagendra Nath Barman, Anjan Jyoti Nath, Sharmita Doley, Shameem Ara Begum, Parikshit Kakati, Sailendra Kumar Das, Taibur Rahman, Dipak Bhuyan, Bhaben Chandra Baishya, Susanta Goswami

**Affiliations:** 1 Department of Microbiology, Faculty of Veterinary Science, Assam Agricultural University, Khanapara, Guwahati, Assam, India; 2 Department of Microbiology, Lakhimpur College of Veterinary Science, Assam Agricultural University, Joyhing, North Lakhimpur, Assam, India; 3 Department of Pathology, Faculty of Veterinary Science, Assam Agricultural University, Khanapara, Guwahati, Assam, India; 4 Department of Parasitology, Faculty of Veterinary Science, Assam Agricultural University, Khanapara, Guwahati, Assam, India; 5 Teaching Veterinary Clinical Complex, Faculty of Veterinary Science, Assam Agricultural University, Khanapara, Guwahati, Assam, India

**Keywords:** antibiogram, Assam, goat, *Listeria monocytogenes*, polymerase chain reaction

## Abstract

**Background and Aim:**

Listeriosis in food animals bears a significant threat to human health. Detailed investigations into the cause facilitate proper management of the disease. This study reports the cultural, pathological, and molecular characterization of *Listeria monocytogenes* isolated from encephalitic goats from peri-urban Guwahati, Assam.

**Materials and Methods:**

Out of nine suspected samples, five positive isolates of *L. monocytogenes* were subjected to bacteriological, biochemical, and molecular tests. The genus and species-specific *L. monocytogenes 16S rRNA* and *prs* genes were amplified by polymerase chain reaction (PCR) to yield 1200 and 370 bp sized products, respectively. The encephalitic form of the disease was characterized by circling movement, high fever, and terminal recumbence.

**Results:**

All the five isolates were confirmed to be *L. monocytogenes* based on PCR amplification of genus and species-specific *16S rRNA* and *prs* gene products. The isolates were sensitive to ciprofloxacin, oxytetracycline (OTC), and norfloxacin, but resistant to doxycycline and erythromycin. A high dose of OTC was used in a goat at the early stage of clinical symptom and the animal recovered clinically.

**Conclusion:**

Listeriosis in goats could pose a significant public health threat as the meat (occasionally milk) or meat products from goats are widely consumed by the people of Assam. Understanding the molecular epidemiological aspects of *L. monocytogenes* infections of food animal species should, therefore, be the priority in this part of the country.

## Introduction

Listeriosis is a disease most frequently encountered in small ruminants such as sheep, goat, as well as in cattle [1,2]. These wide varieties of vertebrates can develop subclinical infections and shed *Listeria* in feces. Clinical cases are seen most often in cattle, sheep, and goats, but they have also been reported in other ruminants [3]. It is an infectious and fatal disease of animals, birds, fish, crustaceans, and humans where septicemia and encephalitis are frequently observed [4]. It is caused by the pathogenic species of Gram-positive, facultative anaerobic intracellular bacteria of the genus *Listeria*. Till now, 17 species of the genus have been identified [5,6]. Vestibular ataxia, circling, head tilt, and unilateral cranial nerve deficits are common meningoencephalitis form of the disease seen in small animals such as sheep and goat [7].

*Listeria monocytogenes* is ubiquitous and widely distributed in nature [8] and has been isolated worldwide from humans, animals, and poultry, environmental sources such as soil, river, and decaying plants, and food sources such as milk, meat and their products, and seafood [4]. *Listeria* species are most commonly found in raw foods, vegetables contaminated by soil and water carrying bacteria, and in raw animal products such as meat and cheese [9]. *L. monocytogenes* can also be isolated from fecal samples of apparently healthy animals, poorly stored silage, and rotten vegetables.

The present report reflects on isolation, molecular characterization, and histopathological alterations due to *L. monocytogenes* infection in goats in a peri-urban locality in the Kamrup district of Assam.

## Materials and Methods

### Ethical approval and informed consent

The study involved clinical cases presented in the Teaching Veterinary Clinical Complex of Faculty of Veterinary Science, Khanapara and informed written consent of the owners were obtained. Animals were handled in compliance with the established ethical regulations and guidelines of IAEC of the university.

### Case description

**Figure-1:**
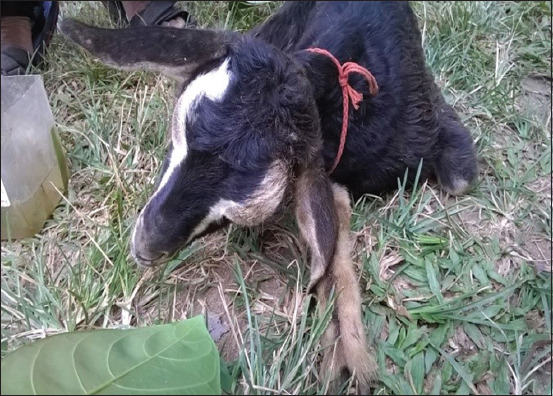
A goat showing a characteristic symptom of head tile.

**Figure-2:**
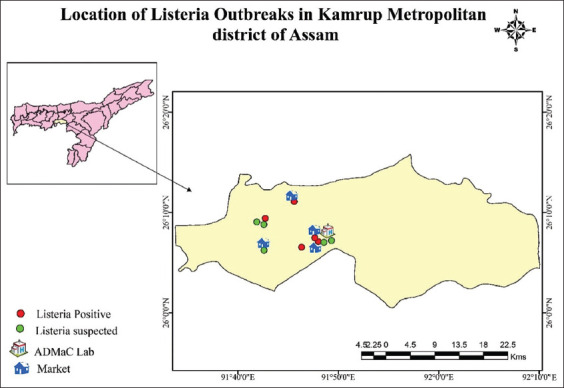
The distribution of *Listeria* positive and suspected cases in the Kamrup Metropolitan district of Assam (Source: Generation of map using ArcMap software 10.2 version).

From November 2016 to October 2017, a few cases (n=10) of goat with neurological disorders had been reported from the peri-urban areas of Kamrup metros of Assam. Affected animals exhibited typical signs of head tilt (Figure-1), fever, and terminal recumbency were brought to the Teaching Veterinary Clinical Complex, College of Veterinary Science, Khanapara, Guwahati, Assam. The detailed microbiological investigation was carried out in the Department of Veterinary Microbiology, College of Veterinary Science, Khanapara, Guwahati, Assam. The distribution of the cases of the outbreak is depicted in Figure-2. The animals were raised in conventional pens or with a raised platform used as a shelter during night and allowed for free grazing during the daytime. This freely moving grower to adult goats picked up leftover foods and vegetable waste from the market as well as roadside areas. Goats were never vaccinated and no deworming was done.

### Laboratory investigation

Blood, serum, and cerebrospinal fluid (CSF) samples were collected from clinically affected animals. In dead animals, a postmortem examination was done and gross changes in different organs, including the brain were recorded. Representative tissue samples such as a lymph node, spleen, liver, and brain tissues were collected for isolation and demonstration of nucleic acid in PCR. Duplicate tissue samples were collected in 10% formalin and processed as per the method described in Luna [10].

For bacterial isolation, brain samples from the dead animals and CSF from the suspected clinically affected animals were collected in a sterile container for isolation of organisms. Tissue samples were aseptically inoculated into brain-heart infusion (BHI) broth (BHI), homogenized for 2 min at room temperature, and incubated at 37°C for 24 h. Simultaneously, brain tissues were subjected for “cold enrichment” by keeping the inoculated broth (with 10% suspension) at 4°C and a loopful of the broth was streaked onto the surface of *Listeria* selective agar (HiMedia, India) containing supplement and incubated at 37°C for 24 h. Typical *Listeria* colonies were purified on nutrient agar. Morphological, biochemical characterization, and the CAMP test were done following the methods described in Cruickshank *et al*. [11] and Cowan and Steel [12]. Isolated organisms were subjected to PCR confirmation.

Freshly isolated cultures of *L. monocytogenes* were subjected to antimicrobial susceptibility tests using the standard protocol. Antibiotics such as amoxicillin (AMX, 10 μg), amoxiclav (AMC, 30 μg), ceftriaxone (30 μg), ciprofloxacin (CIP, 5 μg), doxycycline (Do, 30 μg), erythromycin (E, 10 μg), gentamicin (GEN, 10 μg), norfloxacin (NX, 10 μg), and oxytetracycline (OTC, 30 μg) were used to identify antimicrobial sensitivity pattern. The zone of inhibition was measured, recorded, and interpreted following the guidelines of the Clinical and Laboratory Standards Institute criteria [13].

### Polymerase chain reaction

Amplification of *16S rRNA* (genus-specific) and *prs* gene (species-specific) of *L. monocytogenes* was carried out using the primers and thermocycler conditions as listed in Table-1 [14,15]. The amplified gene products were separated in 1.2% agarose gel and stained with ethidium bromide (0.5 μg/ml). At the same time, samples were processed for an association of peste des petits ruminants virus and Japanese encephalitis virus following the PCR methods described by Widmar *et al*. [14] and Doumith *et al*. [15], respectively.

**Table-1:** Polymerase chain reaction primers and conditions used for amplification of target genes of *Listeria monocytogenes.*

Polymerase chain reaction conditions and primers	Target gene	

	*16S rRNA*	*Prs*
Primer sequence	F: 5’- GACCGGGGCTAATACCGAATGATAA-3’ R: 5’- TTCATGTAGGCGAGTTGCAGCCTA-3’	F: 5’- GCTGAAGAGATTGCGAAAGAAG-3’ R: 5’- CAAAGAAACCTTGGATTTGCGG-3’
Amplicon size (bp)	370	1200
Initial denaturation	94°C, 4 min	94°C, 4 min
Denaturation	94°C, 30 s	94°C, 40 s
Annealing	56°C, 20 s	53°C, 75 s
Extension	72°C, 1 min	72°C, 75 s
Final extension	72°C, 5 min	72°C, 7 min
No. of cycles	34	35
Reference	Widmar *et al.* [14]	Doumith *et al.* [15]

## Results

### Encephalitis in peri-urban goats

Out of 10 clinically affected animals, *L. monocytogenes* infection was confirmed in 5 animals (50.0%) based on clinicopathological changes, microbiological demonstration, and PCR confirmation. Two out of five positive samples (40%) belonged to the age group 6 months-2 years. The other age groups, namely, 0-6 months (n=1, 20%), 2-4 years (n-1, 20%), and more than 4 years (n=1, 20%) were also found to be affected. One out of 5 clinically affected animals of age group 6-month age-old goat was responded to antibiotic treatment. The affected animals exhibited clinical symptoms such as the high rise of temperature (up to 42°C) in initial phase followed by anorexia, profuse salivation, paralysis of lips with impacted cud in mouth, swelling of upper eyelids and ocular discharge, a flickering of the eyeball, paddling/cycling of limbs, trembling and paralysis, walking in circles and in later stage showed lateral recumbency, drooping of ears, dyspnea, and coughing. The location of affected goats (Figure-2) indicated that animals were let loose during daytime and picked up left out offal from market areas.

### Pathological changes

**Figure-3:**
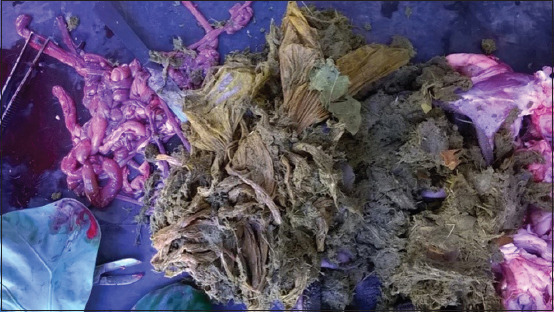
Plastic materials recovered from the rumen.

On postmortem examination, the stomach was impacted by polythene bags (Figure-3) in three out of four dead animals. Besides, there was consistent hemorrhage in the mucosal surface of the abomasum. The brain of the infected animals showed severe congestion. However, no other gross lesions were observed in other organs.

**Figure-4:**
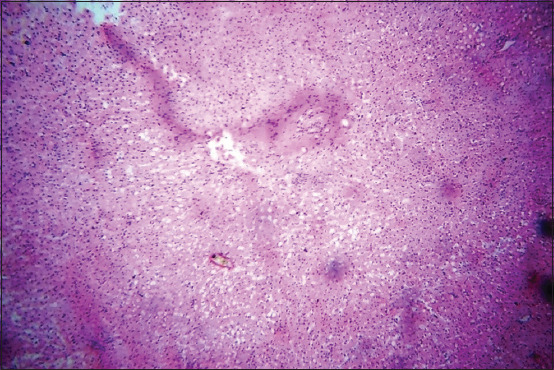
Photomicrograph of the brain showing microabscess H&E, 10×.

**Figure-5:**
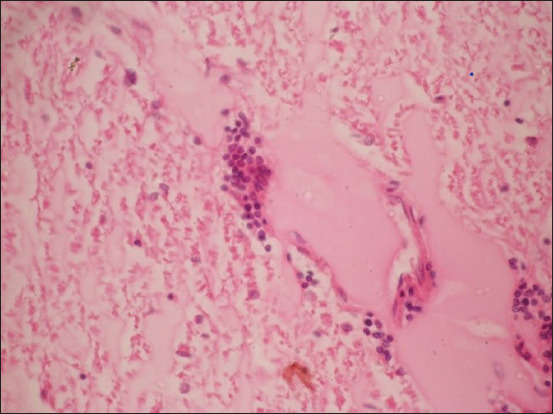
Photomicrograph of the brain showing microabscess with the liquefied necrotic center and mononuclear cell infiltration, H&E, 40×.

Histopathological alterations were restricted to the brain stem (Figures-4 and 5) with microabscesses characterized by focal infiltration of mononuclear cells and a few neutrophils. The microabscesses were centrally liquefied and homogenous in texture. Infiltrating cells giving a perivascular cuffing appearance. In all dead animals, microabscesses with perivascular cuffing were consistently recorded in the brain section.

### Microbiological confirmation

After enrichment and further subculturing into *Listeria* selective agar, small grayish-yellow colonies measuring approximately 1-2 mm in diameter were observed in 24 h aerobic incubation. Microscopically organisms were Gram-positive and coccobacillary in morphology. All the isolates were hemolytic and produced a narrow zone of hemolysis on sheep blood agar. The isolates were catalase, methyl red, and Voges–Proskauer positive but indole and citrate negative.

**Figure-6:**
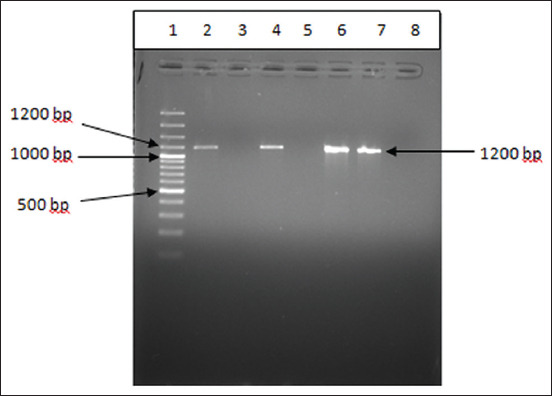
Amplification of the *16S rRNA* gene (1200 bp) of *Listeria monocytogenes* by polymerase chain reaction assay.

**Figure-7:**
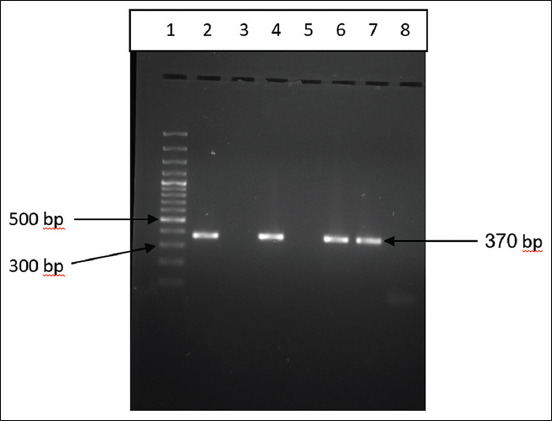
Amplification of *prs* gene (370 bp) of *Listeria monocytogenes* by polymerase chain reaction assay.

The PCR amplified products for *16S rRNA* and *prs* genes, which are genus and species-specific, yielded gene products of 1200 bp and 370 bp size in 1.2% agarose gel (Figures-6 and 7).

Summary of the result of the antibiotic susceptibility pattern of *L. monocytogenes* isolates is shown in Table-2. In general, all the isolates were sensitive to CIP, NX, and OTC (100%) followed by a variable sensitivity pattern against GEN, AMX, and AMC. All the isolates were completely resistant to E and Do.

**Table-2:** Antibiotic sensitivity pattern of *Listeria monocytogenes* isolates from clinical cases of listeriosis suspected goats.

S. No.	Lab. ID	Antibiotics used								

		OTC	GEN	Do	AMX	CRO	CIP	AMC	NX	E
1.	ADMaC/As/Goat/004	S	S	R	R	S	S	R	S	R
2.	ADMaC/As/Goat/006	S	S	R	R	S	S	R	S	R
3.	ADMaC/As/Goat/0011	S	S	R	R	S	S	R	S	R
4.	ADMaC/As/Goat/0012	S	R	R	S	R	S	S	S	R
5.	ADMaC/As/Goat/0015	Negative								
6.	ADMaC/As/Goat/0039									
7.	ADMaC/As/Goat/0045	S	R	R	S	R	S	S	S	R
8.	ADMaC/As/Goat/0049	Negative								
9.	ADMaC/As/Goat/0059									
10.	ADMaC/As/Goat/0060									

OTC=Oxytetracycline, GEN=Gentamicin, Do=Doxycycline, AMX=Amoxicillin, CRO=Ceftriaxone, CIP=Ciprofloxacin, AMC=Amoxiclav, NX=Norfloxacin, E=Erythromycin, S=Sensitive, R=Resistant

In most of the cases, death was rapid after the manifestation of clinical signs. Only one goat at the early stage of clinical symptom survived, which received treatment with a high dose of OTC (Zydus Cadila Vet, India) 10 mg/kg body weight administered intravenously for 7 days along with other supportive medications such as fluid therapy and vitamins.

## Discussion

Listeriosis is one of the important bacterial diseases of different animals with zoonotic potential having broad distribution; it has considerable economic significance as it is mainly a foodborne pathogen. *L. monocytogenes* affect a wide variety of animals, including goat, pig, cattle, buffaloes, dogs, horses, chickens, rabbits, and sometimes in human beings [16]. Organisms are widely distributed in the environment, including soil, vegetation, food of animal origin, silage, fecal material, sewage, and water. Infection occurs along with feed through small wounds in lips, oral and nasal mucosae, and through conjunctiva or consumption of milk from the infected mother. Only immunosuppressed animals become ill following infection [4].

The present investigation revealed a high incidence rate of 50.0% from the suspected clinical cases in goats (n=10) and a high mortality rate among the positive animals (4 out of 5). In small animals such as sheep and goats, recovery is rare but can survive with aggressive antibiotic therapy. The open grazing system was followed for all the animals. All the age groups were found to be susceptible to the highest incidence rate observed in the 2-6 months age group (40% of positive cases). This could occur due to the active grazing behavior of the animals in this age group, which move around actively in search of food. This is interesting to note that most of the dead animals had impacted plastic in their rumen. As these animals were reared under the open grazing method, they often moved to the nearby market place and waste dumping grounds, where they used to consume vegetable and kitchen wastes from the locality. A study carried out peri-urban locality at Guwahati, out of 149 samples, 3 (2.01%) samples were found positive for *Listeria* spp. The highest recovery of *Listeria* spp. was recorded in the garbage (3.63%) followed by decaying vegetables (2.08%) [17]. In other studies, high recovery of *Listeria* spp. (33.3%) was reported from vegetables where *L. monocytogenes* was detected in 22.5% of the vegetables in retail level in Malaysia [18] and 15.3% in raw vegetable salads collected from Chile [19]. *L. monocytogenes* were detected in the feedstuffs (33%) in a dairy farm in Paysandu, Uruguay [6].

The prevalence and incidence of *L. monocytogenes* infection in small ruminants such as sheep and goats have been reported from different countries [1,7,20] most commonly during spring and winter season [1]. In India, *L. monocytogenes* has been reported to occur in raw chicken (6.0%), fish meat (4.0%), beef (2.5%), curd (2.0%), and raw milk (1.3%) samples [21]. It has also been isolated from Ganges water, human clinical, and milk samples [5]. Nayak *et al*. [22] reported 9% isolation of different *Listeria* species from various food samples of animal origin. Saikia and Joshi [23] reported 15% isolation of *L. monocytogenes* from poultry meats collected from the markets of all Northeastern states of India. A review of the epidemiology and risk management of Listeriosis in India by Barbuddhe *et al*. [24] depicted the seriousness of the problem in both animal and human populations. *L. monocytogenes* were found in soil and water tank swabs during the Listeriosis outbreak in a sheep farm in Switzerland [25]. However, there is a scanty report of clinical cases in animals from the Northeastern part of India.

In the present study, the isolates were analyzed for hemolytic activity in 5% sheep blood agar. All the strains were found to be hemolytic, producing a narrow, clear, and complete zone of hemolysis. The pathogenic strains of *L. monocytogenes* are hemolytic and the hemolysin of *Listeria* is a 60 kDa thiol-activated exotoxin designated listeriolysin O, which is a polypeptide of 529 amino acids, sharing strong homologies with SLO and pneumolysin [21,26,27].

The conventional diagnostic tests, such as isolation in growth medium and biochemical characterization methods, are laborious and time-consuming. Molecular diagnostic test such as PCR has been increasingly used as a rapid and accurate detection tool for the detection of *L. monocytogenes* infection in clinical samples and food samples. The genus-specific *16S rRNA*, species-specific *prs*, or other virulence genes such as *act A, inlA, inlB, plcB*, and *hlyA* [21,28,29] are often targeted.

The antibiotic sensitivity pattern of *Listeria* isolates is variable. Many often, the isolates of *Listeria* species are multidrug-resistant [28,30], which could be of prime concern in treating the causes of infection in animals. In most acute cases of listeriosis, the animals often die with little time for treatment. In the present investigation, all the isolates were sensitive to CIP, NX, and OTC. The goat that survived in the present investigation was treated with a high dose of OTC for 10 days. The drug susceptibility or resistance pattern and the differences in virulence factors of commonly isolated *Listeria* species from the farm environment indicate the seriousness of the treatment of outbreaks.

The present investigation suggests that the goats reared in the open grazing system in peri-urban areas of Guwahati city of Assam are susceptible to *L. monocytogenes* infection due to their grazing habits, preferably in the young adult group (6 months-2 years). This, in turn, indicates the circulation of pathogenic strains of *L. monocytes* strains in the animals and environment, making the public vulnerable to infection by food and food products derived from such animals. A detailed serotyping and molecular epidemiological study of listeriosis is, therefore, indicated in and around the Metropolitan district of Kamrup, Assam.

## Conclusion

 There is little information on listeriosis in goats in Assam. Considering the significance of pathogenic *Listeria monocytogenes* infection in this important food animal species and its potential public health hazards, the study demonstrated etiological association, pathology and molecular diagnostic aspects of encephalitic listeriosis in open-grazed goats of peri-urban Guwahati, Assam. Detailed molecular epidemiological studies can provide further insights into the actual disease burden and it will be helpful to adopt adequate managemental strategies to prevent and control the disease.

## Authors’ Contributions

NNB, SKD, and TR conceived and designed the study. AJN and SB drafted the manuscript under the supervision of NNB, SKD, and TR. SD and PK designed the experiment protocol under the supervision of NNB and SG. SB, SKD, PK, DB, and BCB collected and analyzed the samples. AJN and SAB revised the manuscript under the supervision of NNB. All authors read and approved the final manuscript.
